# Assessment of the effects of phenanthrene and its nitrogen heterocyclic analogues on microbial activity in soil

**DOI:** 10.1186/s40064-016-1918-x

**Published:** 2016-03-05

**Authors:** Ihuoma N. Anyanwu, Kirk T. Semple

**Affiliations:** Lancaster Environment Centre, Lancaster University, Lancaster, LA1 4YQ UK; Department of Biological Sciences, Federal University Ndufu-Alike Ikwo, P.M.B 1010, Ebonyi State, Nigeria

**Keywords:** Bioavailability, Ecotoxicity, Microbial activity, Nitrogen-containing PAHs, Phenanthrene, SIR

## Abstract

Microbes are susceptible to contaminant effects, and high concentrations of chemical in soil can impact on microbial growth, density, viability and development. As a result of relative sensitivity of microbes to contaminants, toxicity data are important in determining *critical loads or safe levels* for contaminants in soil. Therefore the aim of this study was to assess the impact of phenanthrene and the 3-ring nitrogen-containing polycyclic aromatic hydrocarbons (N-PAHs) on soil microbial respiration. Soil samples were amended with phenanthrene and its 3-ring nitrogen-containing analogues and respiration rates (using substrate induced respiration), CO_2_ production inhibition and/or stress and total culturable microbial numbers were measured over a 90 days soil-contact time. The study showed that inhibition of phenanthrene amended soils occurred in the first 60 days, while the nitrogen-containing analogues impacted on respiration with increased concentration and contact time. Time dependent inhibitions were more than 25 % portraying N-PAHs toxic and inhibitory effects on microbial synthesis of the added carbon substrate. Further, statistical analysis of data revealed statistically significant differences in the respiration rates over time (p < 0.05). This suggests that soil microorganisms may be more sensitive to N-PAHs in soil than the homocyclic PAH analogues. This current study provides baseline toxicity data to the understanding of the environmental impact of N-PAHs, and assists science-based decision makers for improved management of N-PAH contaminated sites.

## Background

Soil is a complex microhabitat, supporting diverse microbial populations, which play an important role in breakdown and transformation of organic matter in fertile soils with many species contributing to different aspects of soil fertility (OECD 2000). Microbial uptake and conversion of chemicals is continuously taking place throughout the biosphere and it is widely known that indigenous microflora which utilizes organic contaminants in soil as carbon/energy sources are ubiquitous in the environment (Leung et al. 2007). Any interference with these biochemical processes may potentially affect nutrient cycling and impact the health and fertility of soil (OECD 2000).

Phenanthrene and its nitrogen-containing polycyclic aromatic hydrocarbons (N-PAHs) consist of three aromatic rings containing carbon with one or two atoms of nitrogen. These chemicals are semi-volatile, persistent, toxic, ubiquitously distributed (Švábenský et al. 2009; EC 2011; Anyanwu et al. 2013), and are widely produced by industrial activities (petroleum derived, combustion sources and biological sources) (Švábenský et al. 2009; Hazardous Substance Data Bank 2010; EC 2011; Anyanwu and Semple 2015a). Due to their widespread distribution in the environment, and their physico-chemical properties, they are potentially carcinogenic, mutagenic, teratogenic and genotoxic (Bleeker et al. 2002; EC 2011; IARC 2012; Anyanwu and Semple 2015a, b, c). It has been reported that not only homocyclic aromatic compounds, but also heterocyclic compounds contribute to the changes in microbial activity in soil (Anyanwu and Semple 2015a, b). From both toxicological and epidemiological studies, many heterocyclic aromatics have shown to be highly toxic (Hazardous Substance Data Bank 2010; EC 2011). Although the available published data are limited, there are considerable evidence indicating their toxicity to humans and ecological receptors (Bleeker et al. 2002; Hazardous Substance Data Bank 2010; EC 2011; Brar et al. 2010; IARC 2012; Anyanwu et al. 2013; Anyanwu and Semple 2015a, b, c).

It has been reported that microbes are susceptible to contaminant effects; and high concentrations of chemical in soil can impact on microbial growth, density, viability and development (Welp and Brümmer 1999). As a result of relative sensitivity of microbes to contaminants, toxicity data are important in determining *critical loads or safe levels* for contaminants in soil; because, protecting the ecosystem community also protects its functions and ecosystem services (SETAC 2012). In assessing the toxicity of chemicals in soil, various procedures must be taken into consideration. A number of bioassays have been developed to assess contaminant toxicity in soil such as: activity of a range of enzymes, C and N mineralisation and nitrification (Welp and Brümmer 1999; Gong et al. 2000; Thiele-Bruhn and Beck 2005; Butler et al. 2011; Pietravalle and Aspray 2013). However, assessing the impacts of a chemical in soil should also focus on the activity and respiration of microbial populations responsible for carbon transformation, since it subjects them to starvation, changes in the community-level physiological profile, chemical stress/inhibition and death (Domsch et al. 1983; Nwachukwu and Pulford 2011; Butler et al. 2011; Fahrenfeld et al. 2013). Microbial respiration therefore is an effective measure of rate of carbon mineralization, since about 70 % carbon added to soil is lost mainly as CO_2_ and H_2_O, a product of microbial respiration (Usman et al. 2004). Furthermore, evaluating toxicity may be detected in soil in which an easily metabolisable substrate (e.g. glucose) has been added (Meyers et al. 2007; George et al. 2008). Following this, any impact of contaminants may be recorded as changes in the rate and extent of CO_2_ production (OECD 2000; Nwachukwu and Pulford 2011).

Thus, substrate induced respiration (SIR) is a measure of the CO_2_ production from a soil sample after administering an optimal concentration of an additional readily utilizable carbon source. SIR, using glucose, is an indirect and simple method of estimating microbial activities to chemicals in soil. Although some bioassays use other methods for quantifying respiration rate, the most common criterion is CO_2_ released (µg CO_2_ h g^−1^ soil) or O_2_ consumed (µg O_2_ h g^−1^ soil) (Nwachukwu and Pulford 2011; Pietravalle and Aspray 2013).

Microbes are ecological receptors mentioned in recent reviews as requiring much research attention due to their sensitivity to contaminants. Irrespective of this, studies have focused on metals (Bardgett and Saggar 1994), PAHs (Towell et al. 2010), trinitrotoluene (George et al. 2008; Butler et al. 2011; Fahrenfeld et al. 2013), diesel (Sutton et al. 2013), total petroleum hydrocarbons (Pietravalle and Aspray 2013) and other persistent organic pollutants (Welp and Brümmer 1999). However, there is general lack of information on the impact of N-PAHs on soil microbial respiration. Therefore, the aim of this study was to assess the impact of phenanthrene and its 3-ring N-PAHs on soil microbial respiration. With an automated respirometer, it is possible to obtain real time measurement of the CO_2_ production in many samples after the addition of glucose supplement. Thus, SIR using standard laboratory equipment the “Automated Columbus Instrument’s Micro-Oxymax” was used for this study because it is capable of measuring the production of CO_2_ from soil over time. In addition, chemical analysis was performed to measure the loss of contaminants in soil over time.

## Methods

### Chemicals

Phenanthrene (Phen), 1,7-phenanthroline (1,7-Phen), 4,7-phenanthroline (4,7-Phen), ^12^C-glucose and benzo[h]quinoline (B[h]Q) were purchased from Sigma-Aldrich Company Ltd, UK.

### Soil preparation

An agricultural soil was collected from the top layer of field under pasture from a depth of approximately 5–20 cm from Myerscough Agricultural College Lancashire, UK. The soil was sandy loam (19.5 % clay, 60.4 % sand, 20.0 % silt) with an organic matter content of 2.7 % and pH 6.5 (Table 1). The soil was air dried at room temperature and then sieved through a 2 mm sieve to remove roots and stones. Prior spiking, the soil was rehydrated with deionised water back to original water holding capacity (WHC). The soils were amended with phenanthrene and N-PAH standards dissolved in acetone using the method described by (Doick et al. 2003). Soils were placed in bowls and ^1^/_3_ of the soil (100 g) was amended with dissolved chemicals to give concentrations of 10, 100, 250 and 500 mg kg^−1^. The carrier solvent (acetone) was left to evaporate for 4 h from the soil, after which it was mixed with the remaining ^2^/_3_ of the soil (200 g) and the final concentrations of test chemicals in the obtained soil sample were measured (Table 2). Analytical blanks were prepared using soils amended with acetone only to serve as control. The soils were then put in amber jars and incubated in the dark at 21 ± 1 °C for 90 days.

**Table 1:** Myerscough soil characteristics (n = 3), adapted from Couling et al. (2010)

Soil characteristics	Parameter value
pH (in dH_2_O) (n = 5)	6.50 ± 0.08
Field moisture content (%) (n = 3)	21.07 ± 2.78
Microbial heterotrophic numbers (CFUg^−1^)	2.17 × 105 ± 1.67 × 104
Element analysis (n = 10)	
Total extractable carbon	1.8 % ± 0.03
Total extractable nitrogen	0.14 % ± 0.01
Total extractable organic carbon	1.6 % ± 0.07
Soil organic matter	2.7 % ± 0.04
Phosphorus (μg g^−1^)	997.00 ± 0.01
Soil particle properties (n = 3)	
Clay	19.50 % ± 0.70
Silt	20.00 % ± 0.87
Sand—Total	60.40 % ± 1.20
Coarse sand	0.12 % ± 0.01
Medium sand	6.90 % ± 0.10
Fine sand	53.30 % ± 0.60
Surface texture: sandy loam

**Table 2:** Final concentrations of test chemicals in the obtained soil sample

Compound	Initial concentration (mg kg^−1^)	Final concentration (mg kg^−1^)	Recoveries (%)
Phenanthrene	10	6.70	67.00
	100	78.00	78.00
	250	241.00	96.40
	500	494.50	98.90
1,7-Phenanthroline	10	4.90	49.00
	100	59.30	59.30
	250	238.00	95.20
	500	523.60	104.70
Benzo[h]quinoline	10	7.00	70.00
	100	59.20	59.20
	250	227.10	90.80
	500	504.50	100.90
4,7-Phenanthroline	10	7.80	78.00
	100	91.90	91.90
	250	235.80	94.30
	500	408.60	81.70

### Substrate-induced respiration

Microbial respiration assay was performed according to OECD guideline draft 217 (2000). Samples were analyzed after 0, 30, 60, and 90 days soil-contact time. At each time point 10 g of soil (in triplicate per treatment) were placed in 250 ml Duran^(R)^ bottle and amended with glucose substrate (5 mg g^−1^ dissolved in sterile deionized water). The glucose concentration was determined based on preliminary work. The glucose solution was added to the Duran bottles and connected to an automated Columbus Instrument’s Micro-Oxymax respirometer, which connected to a computer and used to process data from the CO_2_ sensor. The system was set to collect data bihourly for 12 h incubated at 21 ± 1 °C. Following data collection, the percentage inhibition and/or stress of the PAH and N-PAHs on CO_2_ production were calculated to determine changes in the environment, using the formula:

$$\% \, inhibition = 1 - \frac{b}{c} \times 100$$

where b is the mean cumulative CO_2_ in amended soil samples; c is the mean cumulative CO_2_ in un-amended soil samples (OECD 2000; Nwachukwu and Pulford 2011).

### Enumeration of culturable microbial numbers

Enumeration of the total culturable microbial numbers was determined by a colony forming unit count (CFUs g^−1^ soil). This was performed at the start of each time point in non-glucose amended soils. Soil samples (1 g) were mixed with 9 ml of Ringer’s solution by whirl-mixing for 1 min and allowed to stand for 3 min. Soil solution (0.1 ml) was serially diluted in 0.9 ml Ringers solution; aliquots (0.0 l ml) of these dilutions were spread on plate count agar (PCA). CFUs were counted after 2 days of incubation.

### Solvent extraction and analysis of chemicals

Samples from each amended soil (5 g) in triplicate per treatment were mixed with 5 g sodium sulphate and placed in glass centrifuge tubes (35 ml capacity) to which 25 ml mixture of hexane/ethyl acetate (80:20) was added. The tubes were sealed and place on their sides on an orbital shaker (Janke and Kunkel, IKA^®^-Labortechnik KS 250) and shaken at 150 rpm for 20 h. The tubes were then centrifuged at 800 rpm for 20 min (AccuSpin™ 1, Fisher Scientific). The supernatant (5 ml) was taken and concentrated to 1 ml, after which the extract was cleaned-up using a 5 mm column containing 4 g of 2 % water deactivated aluminum-oxide topped with 1 g of sodium sulphate. Elution was achieved using 30 ml hexane/ethyl acetate (1:1). The eluent was concentrated to 1 ml under a stream nitrogen and analyzed with the ThermoQuest Trace GC Finningan Trace MS with CP-Sil 8CB (in full scan) using 50 m × 0.25 mm. 0.12 µm, Varian column. The injector type was SSL, split less injection, source temperature was 250 °C, oven temperature was programmed for 70 °C (2 min), 10 °C min^−1^ to 150 °C, 4 °C min^−1^ to 250 °C, held for 10 min. Ion source was EI+, MS interface temperature was 300 °C, the electron energy was >−70 eV, emission current was 300 µA and resolution was 1 amu. Mass range (m z^−1^) was 120–300 and the scan rate was 2.5 scan s^−1^. Quantification was performed on the absolute calibration curve method in the range of 1–8 µg ml^−1^. Deuterated standard of acridine (2 µg ml^−1^) was used as internal standard. The masses of the parent compound are compared with the largest mass peak in the spectra. For quality assurance, all solvents used for the analysis were HPLC grade and the glassware’s were soaked overnight in deacon, rinsed, dried and then washed in acetone. The recoveries for the test chemicals ranged from 49.5 to 104.7 % (0 day), 22.0 to 113.7 % (30 days), 6.0 to 93.4 % (60 days) and 2.0 to 87.5 % (90 days).

### Data analysis

The mean, standard deviation and standard error of the extractible concentrations, cumulative CO_2_ production and CFUs were calculated. Distribution fit for normality test using Shapiro–Wilk was performed. Statistical analyses were carried out with linear regression of the SPSS 20 package. Mean CO_2_ production in control soils was used as dependent variables while amended soils were used as independent variables. Analysis of Variance (ANOVA) was used to determine the statistical significance difference in mean CO_2_ among treatments and incubation periods (p < 0.05). Data was presented as mean ± SE and graphs were plotted using SigmaPlot 10.0 version.

## Results

### Removal of phenanthrene and 3-ring N-PAHs from soil over time

**Fig. 1:**
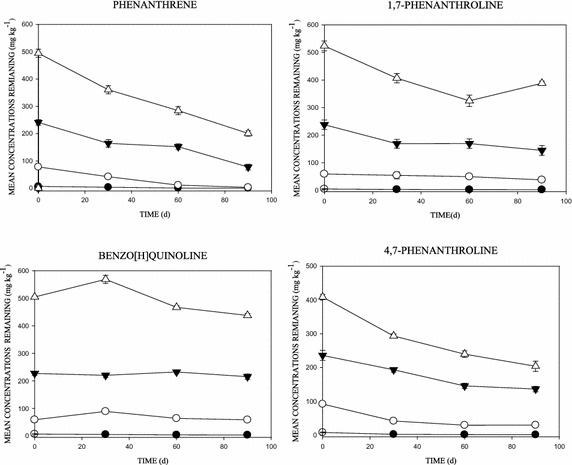
Chemical transformation over time in soils amended with different concentrations of phenanthrene and the 3-ring N-PAHs. Data shows: 10 mg kg^−1^ (*filled circle*), 100 mg kg^−1^ (*open circle*), 250 mg kg^−1^ (*filled down-pointing triangle*) and 500 mg kg^−1^ (*open triangle*)

The removal of phenanthrene and similar 3-ring N-PAHs was measured in the soil over 90 days (Fig. 1). Although not consistent, it was observed that as soil contact time increased, the concentrations of the aromatics declined. In samples amended with 10 mg kg^−1^, low concentrations of the chemicals remained after 30 days, especially in the phenanthrene amended soils. Consequently, at 90 days, only 2 % (10 mg kg^−1^), 3.1 % (100 mg kg^−1^), 31 % (250 mg kg^−1^) and 40.1 % (500 mg kg^−1^) of the phenanthrene remained in the amended soils (p < 0.05) (Fig. 1). However, in the N-PAH amended soils, 22 % (10 mg kg^−1^), 38 % (100 mg kg^−1^), 57.9 % (250 mg kg^−1^) and 77.6 % (500 mg kg^−1^) for 1,7-Phen, and 38 % (10 mg kg^−1^), 59 % (100 mg kg^−1^), 86.2 % (250 mg kg^−1^) and 87.5 % (500 mg kg^−1^) for B[h]Q was measured (p < 0.05); with the exception of 4,7-phenanthroline which seemed to decline faster than other N-PAHs; with values of 11 % (10 mg kg^−1^), 29.6 % (100 mg kg^−1^), 54.5 % (250 mg kg^−1^) and 40.8 % (500 mg kg^−1^) (Fig. 1).

### Substrate induced respiration

**Fig. 2:**
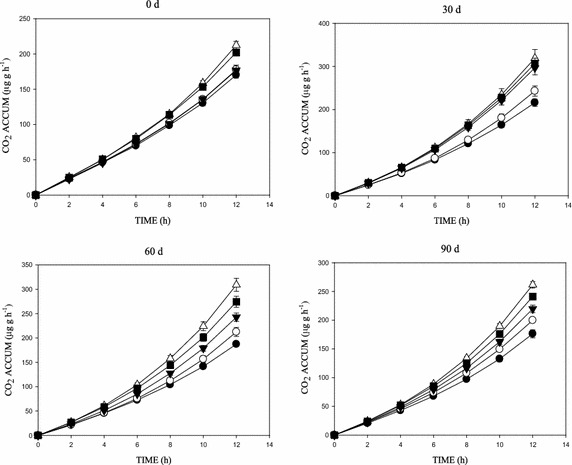
Cumulative CO_2_ production in phenanthrene amended soil following amendment with glucose substrate during the 0–90 days incubation period. Data shows: 0 mg kg^−1^ (*filled square*), 10 mg kg^−1^ (*open triangle*), 100 mg kg^−1^ (*filled down-pointing triangle*), 250 mg kg^−1^ (*open circle*) and 500 mg kg^−1^ (*filled circle*)

**Fig. 3:**
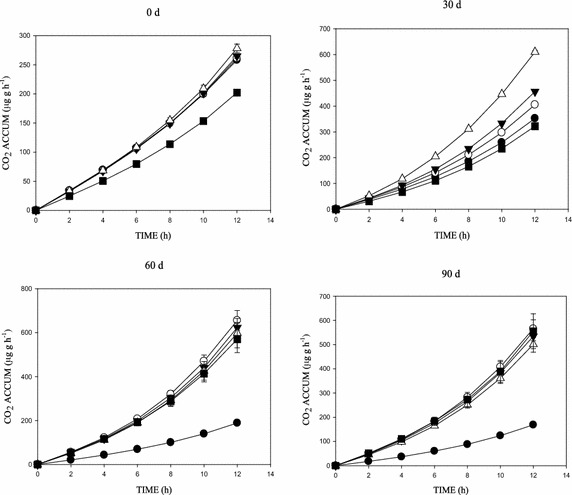
Cumulative CO_2_ production in 1,7-phenanthroline amended soil following amendment with glucose substrate during the 0–90 days incubation period. Data shows: 0 mg kg^−1^ (*filled square*), 10 mg kg^−1^ (*open triangle*), 100 mg kg^−1^ (*filled down-pointing triangle*), 250 mg kg^−1^ (*open circle*) and 500 mg kg^−1^ (*filled circle*)

**Fig. 4:**
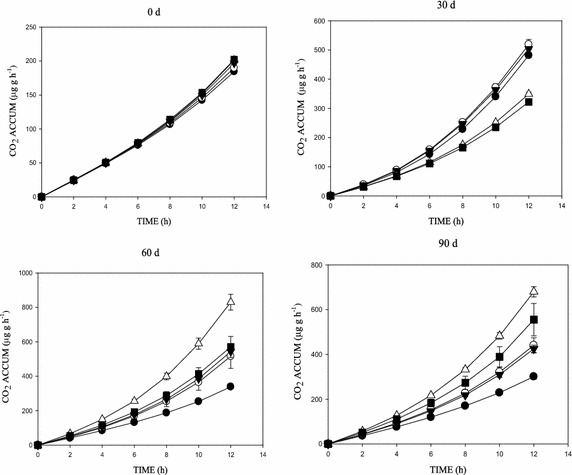
Cumulative CO_2_ production in benzo[h]quinoline amended soil following amendment with glucose substrate during the 0–90 days incubation period. Data shows: 0 mg kg^−1^ (*filled square*), 10 mg kg^−1^ (*open triangle*), 100 mg kg^−1^ (*filled down-pointing triangle*), 250 mg kg^−1^ (*open circle*) and 500 mg kg^−1^ (*filled circle*)

**Fig. 5:**
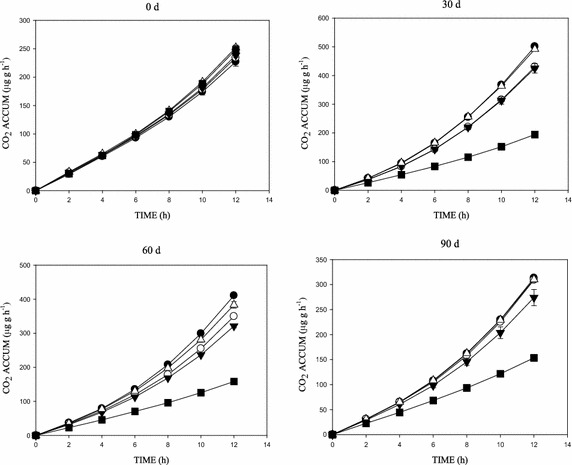
Cumulative CO_2_ production in 4,7-phenanthroline amended soil following amendment with glucose substrate during the 0 – 90 d incubation period. Data shows: 0 mg kg^−1^ (*filled square*), 10 mg kg^−1^ (*open triangle*), 100 mg kg^−1^ (*filled down-pointing triangle*), 250 mg kg^−1^ (*open circle*) and 500 mg kg^−1^ (*filled circle*)

Figures 2, 3, 4 and 5 shows the concentration effects of phenanthrene and the 3-ring N-PAHs on soil microbial respiration over time. SIR, measured as CO_2_ production, showed significant increases in respiration following the addition of glucose, with the CO_2_ increases generally higher in the lowest concentration (10 mg kg^−1^) than the control soil (Figs. 2, 3, 4, 5). There were variations in the mineralisation of glucose between the aromatics. Also, increase in CO_2_ production was observed with increased substrate-contact time. In addition, the cumulative CO_2_ production vs time (h) graph showed an inhibition and/or stress in the biotic decomposition of the added carbon substrate (Figs. 2, 3, 4). Further, the 0–90 days concentration related impact of phenanthrene and the 3-ring N-PAH analogues on microbial activity in soil followed a predictable pattern of mild inhibitions at 0 days, which changed after 30 days (Figs. 2, 3, 4, 5). Furthermore, a clear concentration–time-effect was observed among the chemicals (p < 0.05). Although showing much variability by comparison; there was a noticeable pattern of an initial relapse in CO_2_ production, followed by a recovery, and then inhibition in the presence of the chemicals; this initial inhibition was most evident for the samples amended with the highest concentration of 500 mg kg^−1^ (Figs. 2, 3, 4). For example, in phenanthrene amended soils, there was a concentration related effect at the higher concentrations in the first 60 days of incubation. However, inhibitory effects were recorded in the 500 mg kg^−1^ (1,7-Phen and B[h]Q) amended soils throughout the 90 days incubation, with the exception of the 4,7-Phen amendments which showed no inhibition (Fig. 5). Among the N-PAHs, B[h]Q exhibited a trend of increased inhibition in the 100, 250 and 500 mg kg^−1^ amendments with increase in soil-contact time (Fig. 4). Analysis of data between the variables (mean CO_2_ production across treatments and incubation periods) using ANOVA showed a statistically significant difference in all the treatments, at all the times (p < 0.05).

CO_2_ production rates was measured, and the result showed a trend of decline in respiration rates with increase in chemical concentrations, with the exception of 1,7-Phen and B[h]Q (30 days) and 4,7-Phen (Table 3). Phenanthrene was observed to exhibit consistent decrease in respiration rates with increased soil-contact-time. However, the soils amended with the N-PAHs exhibited a trend of increased CO_2_ production rates with increase in contact time, which declined slightly at 90 days; with the exception of 4,7-Phen. Among the N-PAHs, however, while 1,7-Phen and B[h]Q soils recorded decrease in respiration rates (30 days), increased values was observed in the 4,7-Phen amended soils (Table 3). Statistical analysis of data showed strong positive correlations that are statistically significant among treatments and incubation periods (p < 0.05). However, the 4,7-Phen amended soils showed no statistical significant difference in CO_2_ production rates with incubation (p > 0.05). Also, the distribution fit for normality test (Shapiro–Wilk) fits the normal distribution curve (p > 0.05), so sample normality was assumed.

**Table 3:** Mean CO_2_ production rates (µg h g^−1^) in the phenanthrene and 3-ring N-PAHs amended soils with glucose addition over time

Chemical	Time (d)	Concentrations (mg kg^−1^)				
		0	10	100	250	500
Phenanthrene	0	12.50 ± 0.50	13.70 ± 0.50	10.50 ± 0.80	10.70 ± 0.10	10.20 ± 0.10
	30	20.20 ± 1.00	21.50 ± 1.70	19.20 ± 1.40	15.90 ± 0.70	13.20 ± 0.70
	60	18.60 ± 0.80	21.60 ± 1.10	16.30 ± 0.60	14.30 ± 0.60	11.70 ± 0.30
	90	16.60 ± 0.30	18.30 ± 0.30	14.60 ± 0.50	12.80 ± 0.10	11.10 ± 0.60
1,7-Phenanathroline	0	12.40 ± 0.20	17.60 ± 0.40	16.20 ± 0.40	15.70 ± 0.40	14.90 ± 0.30
	30	2.70 ± 0.30	2.50 ± 0.20	2.60 ± 0.20	3.30 ± 0.40	15.20 ± 2.60
	60	39.10 ± 6.00	42.70 ± 6.00	46.00 ± 2.60	47.10 ± 4.60	12.60 ± 0.80
	90	41.40 ± 6.70	35.50 ± 2.80	38.20 ± 1.90	40.30 ± 3.80	11.40 ± 0.10
Benzo[h]quinoline	0	12.40 ± 0.20	12.60 ± 0.30	12.10 ± 0.10	11.20 ± 0.20	10.70 ± 0.10
	30	2.70 ± 0.30	2.50 ± 0.10	2.60 ± 0.10	2.50 ± 0.10	3.50 ± 0.20
	60	39.10 ± 6.0	60.70 ± 4.10	39.70 ± 0.30	38.20 ± 5.90	21.60 ± 1.50
	90	41.40 ± 6.70	49.80 ± 2.30	29.50 ± 1.30	30.50 ± 2.80	18.30 ± 1.30
4,7-Phenanthroline	0	12.50 ± 0.50	15.50 ± 0.40	14.70 ± 0.30	14.30 ± 0.20	13.50 ± 0.60
	30	10.80 ± 0.10	32.70 ± 0.10	28.60 ± 1.00	29.30 ± 0.50	34.20 ± 0.30
	60	8.30 ± 0.60	26.10 ± 0.50	21.60 ± 0.40	24.20 ± 0.40	28.50 ± 0.20
	90	8.10 ± 0.30	20.60 ± 0.40	17.90 ± 1.20	21.40 ± 0.30	21.30 ± 0.40

Data shows different concentrations (mg kg^−1^) and incubation days, respectively

The inhibition on respiration (%) was calculated (Table 4), and the 0–90 days interaction of chemical with soil showed strong inhibitory effects (>25 %) in the 1,7-Phen and B[h]Q amended soil samples (Table 4). The inhibitory effects on microbial respiration followed a pattern of increased inhibition/stress with incubation. Further, N-PAHs displayed significant chemical inhibition/stress, while chemical inhibition was generally less pronounced in the phenanthrene amended soils over time; however, 4,7-Phen recorded no chemical inhibition (NI) on microbial activity in soil (Table 4). Statistical analysis of data with ANOVA showed strong positive correlations between treatments and incubation times in all the chemicals (p < 0.05).

**Table 4:** Inhibition (%) by phenanthrene and the 3-ring N-PAHs on CO_2_ production in amended soils with glucose addition over time

Chemical	Time (d)	Inhibition (%) by chemical concentration (mg kg^−1^)				
		10	100	250	500	R^2^
Phenanthrene	0	5.00	12.50	12.10	15.70	0.926
	30	3.90	3.20	20.70	29.50	
	60	12.60	11.40	22.50	31.70	
	90	8.40	8.80	17.10	26.60	
1,7-Phenantholine	0	37.60	31.00	29.30	27.70	0.932
	30	89.30	41.70	25.90	9.40	
	60	4.80	9.30	14.90	66.80	
	90	9.80	3.70	1.80	69.90	
Benzo[h]quinoline	0	0.40	2.60	6.00	8.70	0.942
	30	8.40	56.50	61.00	49.50	
	60	45.60	4.60	9.40	40.50	
	90	22.30	23.40	20.60	45.80	
4,7-Phenanthroline	0	1.40	4.00	5.60	8.50	0.989
	30	NI	NI	NI	NI	
	60	NI	NI	NI	NI	
	90	NI	NI	NI	NI	

Data shows different chemical concentrations (mg kg^−1^) and incubation days, respectively

*NI* no inhibition, *R* ^2^ correlation coefficient

### Total culturable microbial numbers

In order to link the observed CO_2_ production with microbial population; the total culturable microbial numbers was measured (Table 5) and described as the mean colony forming units (CFUs) of Phen and N-PAHs amended soils. The mean CFUs did not differ significantly from the control soils (p > 0.05); however, Phen amended soils had significantly higher CFUs than the N-PAHs amended soils with increase in soil contact time (Table 5). The culturable microbial numbers were different in each of the exposure concentration and chemical; with the CFUs recording highest in the lower concentrations. Although not showing a consistent trend, the results showed that increasing N-PAHs (B[h]Q and 1,7-Phen) contamination resulted in reductions in the CFUs (Table 5).

**Table 5:** Total culturable counts (CFUg^−1^) in soils amended with phenanthrene and the 3-ring N-PAHs over time

Chemical	Time (d)	Mean culturable microbial numbers (CFUs g^−1^)				
		Concentrations (mg kg^−1^)				
		0	10	100	250	500
Phenanthrene	0	1.60 × 10^6^	1.25 × 10^6^	0.90 × 10^6^	0.97 × 10^6^	0.64 × 10^6^
	30	2.25 × 10^6^	2.50 × 10^6^	1.90 × 10^6^	1.77 × 10^6^	1.23 × 10^6^
	60	0.98 × 10^6^	0.77 × 10^6^	1.23 × 10^6^	1.33 × 10^6^	1.16 × 10^6^
	90	1.15 × 10^6^	1.5 × 10^6^	1.10 × 10^6^	1.23 × 10^6^	1.20 × 10^6^
1,7-Phenanthroline	0	1.92 × 10^6^	1.25 × 10^6^	0.90 × 10^6^	0.90 × 10^6^	0.90 × 10^6^
	30	1.35 × 10^6^	1.40 × 10^6^	1.60 × 10^6^	1.10 × 10^6^	0.98 × 10^6^
	60	1.03 × 10^6^	0.45 × 10^6^	1.40 × 10^6^	0.32 × 10^6^	0.37 × 10^6^
	90	1.32 × 10^6^	1.50 × 10^6^	1.25 × 10^6^	1.23 × 10^6^	0.73 × 10^6^
Benzo[h]quinoline	0	0.87 × 10^6^	0.65 × 10^6^	0.62 × 10^6^	0.55 × 10^6^	0.62 × 10^6^
	30	0.60 × 10^6^	0.60 × 10^6^	0.78 × 10^6^	0.90 × 10^6^	0.73 × 10^6^
	60	1.02 × 10^6^	1.75 × 10^6^	0.75 × 10^6^	0.53 × 10^6^	0.25 × 10^6^
	90	0.75 × 10^6^	1.40 × 10^6^	0.69 × 10^6^	0.51 × 10^6^	0.30 × 10^6^
4,7-Phenanthroline	0	1.55 × 10^6^	1.00 × 10^6^	0.65 × 10^6^	0.35 × 10^6^	0.39 × 10^6^
	30	0.5 × 10^6^	1.05 × 10^6^	1.00 × 10^6^	0.80 × 10^6^	0.75 × 10^6^
	60	1.01 × 10^6^	1.10 × 10^6^	1.09 × 10^6^	0.78 × 10^6^	0.51 × 10^6^
	90	0.77 × 10^6^	1.00 × 10^6^	0.99 × 10^6^	2.01 × 10^6^	1.49 × 10^6^

Data shows different chemical concentrations (mg kg^−1^) and incubation days, respectively. CFUs = (Total culturable counts per gram of soil in plate count agar)

## Discussion

From the results, it can be seen that increased soil-contaminant-contact time showed removal of chemicals as well as reductions in the extraction of the remaining chemicals in soil; this may be due to volatilization, sorption and/or biodegradation (Welp and Brümmer 1999; Semple et al. 2003; Anyanwu and Semple 2015b). In soil for example, organic contaminants are known to undergo irreversible sorption. Consequently, with both organic matter (2.7 %) and pH (6.5), a significant proportion of chemicals applied may diffuse into soil pores and thus, become occluded and un-available/non-extractible (Semple et al. 2003). Further, Welp and Brümmer (1999) reported that a decrease in the bioavailability/extractability of organic contaminants is mostly due to sorption. In addition, biodegradative processes are known to contribute to loss (Towell et al. 2010; Anyanwu and Semple 2015b). Irrespective of the low concentrations measured, the inhibitory and/or stress effects on soil indigenous microbes were still obvious. This is in agreement with the reports on antibiotics and other organic pollutants in literature (Welp and Brümmer 1999; Thiele-Bruhn and Beck 2005; Anyanwu and Semple 2015b; c).

To stimulate microbial activity, growth and viability, the addition of nutrients to soil is necessary. Also, to enable microbial growth in the presence of a carbon substrate, the SIR time was extended to 12 h (OECD 2000). In this study, the SIR results showed that CO_2_ production was affected by chemical concentrations and incubation times. This is in agreement with the numerous studies in literature that used carbon substrate (Thiele-Bruhn and Beck 2005; Meyers et al. 2007; George et al. 2008) and Fe(III) reduction test (Thiele-Bruhn and Beck 2005; Welp and Brümmer 1999), all of whom reported concentration related toxic effects of organic pollutants on soil microbial activity. This suggests that N-PAHs exerted inhibitory effects on microbial respiration and activity in soil.

From the data, N-PAHs exhibited a trend of increased CO_2_ production rates with increase in contact time; increased CO_2_ production suggests increasing stress on microbial cells and the expending of energy to support cellular survival and/or maintenance could be an indication of ecotoxicological effects. The observed increase in CO_2_ production rates in this study is in agreement with the findings of Sutton et al. (2013) and Pietravalle and Aspray (2013), who reported higher rates of CO_2_ production in soils associated with diesel amendments. Similarly, Bardgett and Saggar (1994) reported increased CO_2_ production with increased chemical concentrations and beyond a threshold of 500 mg kg^−1^, there was severe disruption in the normal functioning of soil microorganisms. Conversely, this current study showed decreases in the cumulative CO_2_ production with increased N-PAH concentrations and persistent inhibition at 500 mg kg^−1^; chemical differences and physico-chemical isomers may be attributable. Further, it could be that the highest concentration (500 mg kg^−1^) was so toxic that the cells were killed thereby reducing CO_2_ production.

The ability of the chemicals to sustain microbial respiration in the presence of toxicity was significant at the lower concentrations, with the exception of 4,7-phen; differences in N-atom position and/or sensitivity of micro-organisms to the chemicals may be attributable. Thiele-Bruhn and Beck (2005) reported that shifts in microbial community structure could compensate for effects on species, in which case the tolerance of the dominant microbial specie determines the respiration. It is possible that at the lowest concentration, the microbes may be compensated by a higher C turnover and so lead to a gradual change in viability. However, at much higher concentrations, the contaminants caused inhibitions, cell death and disruptions in the normal functioning of the microbial community. Further, the higher CFUs observed in phenanthrene amended soils than the N-PAH soils (in this study), may be attributed to biodegradation and/or adaptation to toxicity. For example, it has been reported that when a chemical is added to soil, the microorganisms may have different responses: some may lyse, while others may be resistant to the pollutant thereby resulting in an increase in cell numbers because of reduced competition. In this current study, however, it may be that stress due to nutrient deficits caused the microbial populations to become vulnerable to the toxic chemicals (Gong et al. 2000).

Further, this current study revealed that the presence of N-PAHs changed the response of the microbial activity and function over time. Various authors have made numerous suggestions on the antimicrobial properties and responses of microorganisms to organic contaminants (Domsch et al. 1983; Welp and Brümmer 1999; Gong et al. 2000; Thiele-Bruhn and Beck 2005; Meyers et al. 2007; George et al. 2008; Butler et al. 2011; Fahrenfeld et al. 2013; Anyanwu and Semple 2015b), all of whom reported that trinitrotoluenes, antibiotics, explosives, triclosan and POPs contaminations are known to cause inhibitory effects on soil microbial functions, reduced biological activities, cause dynamic states and shifts in the soil microbial community structure. In addition, Domsch et al. (1983) documented that any alteration caused by either natural agents or pollutants which returns to normal microbiology within 30 days should be considered normal fluctuations; alterations lasting for 60 days can be regarded as tolerable but those persisting for over 90 days are stress agents. Thus, N-PAHs are not only regarded as toxins, but as environmental stressors; indicating that N-PAH contamination may have long term impacts on soil microbial activity. Furthermore, the study identified B[h]Q as the most toxic chemical; because, it exhibited consistent stress/inhibitory effects on soil microbial activity over time; suggesting that the contaminant may persist in soil.

## Conclusions

It can now be clearly understood how severely soil microorganisms and microbial activity can be disrupted by elevated N-PAHs concentrations. The studied effect of phenanthrene and N-PAHs on soil microbial respiration delineates N-PAHs as environmental toxins which are more toxic to microbes than their homocyclic analogues in soil with ageing. The results showed that extent of respiration, inhibition and/or stress revealed N-PAHs bioavailability, toxicity and persistence. However, the effects of other N-PAHs and their bio-transformed metabolites require further investigation.
